# Tactical use of irrigants in open orthopaedic procedures: ‘what to use & when’

**DOI:** 10.1530/EOR-2025-0136

**Published:** 2026-03-02

**Authors:** Madhav Chowdhry, Abdul Qayyum Khan, Christopher Hamad, Nicholas M Bernthal, Edward J McPherson

**Affiliations:** ^1^ Department of Orthopedic Surgery, Jawaharlal Nehru Medical College, Aligarh Muslim University, Aligarh, India; ^2^ Department of Continuing Education, University of Oxford, Oxford, UK; ^3^ Department of Orthopaedic Surgery, David Geffen School of Medicine, UCLA, Los Angeles, California, USA

**Keywords:** orthopaedic lavage, irrigants, orthopaedic wounds, aseptic, acute septic, chronic septic, planktonic microbes, biofilm

## Abstract

- Musculoskeletal infections (MSIs) pose formidable challenges to healthcare resources. Surgical wound irrigation is a crucial step in reducing microbial bioburden in open orthopaedic procedures, yet there remains ambiguity regarding optimal use of lavage agents.
- For tactical selection of irrigants, orthopaedic wounds can be classified into three major categories (aseptic, acute septic and chronic septic) based upon microbial bioload and presence or absence of biofilm upon implanted hardware and/or musculoskeletal tissues.
- Irrigant products can be stratified based upon their modes of action: single modal (dilutional), dual modal (dilutional and chemical) and multi-modal (dilutional with multiple mechanisms).
- Host toxicity is commensurate with increased complexity of an irrigant product. A tailored, stepwise strategy of irrigant selection aligned with wound type is recommended, escalating from simple low toxicity dilutional lavage for low microbial bioloads to potent multi-modal chemical agents for biofilm-laden chronic infections.
- The method of irrigant delivery and selected volume are integral to wound lavage. The difference between methods is the impact pressure to host tissues and the depth of fluid penetration. Higher impact pressures clear adherent bacteria and foreign debris albeit the cost of host tissue damage.

## Introduction

Musculoskeletal infections (MSIs) are challenging to treat, often requiring an extraordinary amount of healthcare resources (1, 2, 3). Residual limb and joint dysfunction can adversely affect a patient’s quality of life and ability to provide family support (4, 5, 6). Strategically, prevention and treatment of infection in operative wounds is a keystone premise in surgical site management. Wound lavage agents, based upon the concept of dilutional, chemical and mechanical mechanisms for microbial elimination, play a significant role in reducing microbial bioburden, facilitating a successful outcome. However, confusion and controversy exist to the appropriate selection of irrigants. This review describes a framework for irrigant selection in open (non-arthroscopic) orthopaedic procedures, describing a proportionate use approach, focussing on irrigant mechanism(s) of action, antimicrobial efficacy and host toxicity. This manuscript is not an exhaustive review of all potential irrigants. Rather, the intent is to review wound irrigants currently available to the global orthopaedic community. Tactically, a progressive escalation of irrigant strength commensurate with microbial bioload and biofilm state is required to balance microbial bioburden with host toxicity.

## Stratification of orthopaedic wounds

Orthopaedic wounds can be classified into three major categories: aseptic, acute septic and chronic septic. In aseptic conditions, the surgical wound is realistically inoculated with microbes. Microbes are disseminated and delivered into the surgical field via fomites, the majority of which are skin squamous cells carrying microbes, shed by operating room personnel and patient (7, 8, 9). These fomites are carried by vortex air currents within the operative theatre and delivered into the surgical wound and onto surgical equipment (7, 8, 10). Fomites also include hair with associated microbes and contaminated inanimate particles (e.g. particles from overhead surgical lights) that are inadvertently introduced into the surgical field. Realistically, aseptic wounds contain a low microbial load with microbes in a planktonic state. In this setting, when host immune surveillance is muted (e.g. diabetes, autoimmune conditions and medications) or the surgical technique is not sufficiently strict, the microbial inoculum flourishes with the low-load state progressing into an overt septic condition. Therefore, a gentle irrigation of the surgical wound to reduce microbial load at the termination of the procedure is warranted. In the transition to an acute septic state, microbes establish a threshold colonization, rapidly replicating and eliciting a host inflammatory reaction manifested by overt erythema and gross pus. The wound has a high microbial load with microbes existing in a planktonic state with sessile microbes adhering to tissue surfaces (11, 12, 13). In this condition, an irrigant effective against planktonic and sessile microbes is required to reduce bioload during debridement. Given further time and in the presence of implanted hardware and/or damaged host tissue, the acute septic condition, if left untreated, transforms into a chronic septic condition with the formation of a biofilm. A biofilm can establish as early as 72 h (14, 15, 16). A biofilm is a complex species-specific biomatrix created when microbes adhere onto foreign material surfaces, avascular bone or damaged musculoskeletal tissue (3, 17). It consists of an extracellular polymeric substance (EPS) within which microbes exist and communicate with one another via several signalling mechanisms (18, 19, 20). The EPS, acting as a mechanical barrier, makes it difficult for immune cells and antimicrobial agents to reach and extinguish microbial reserves (21, 22). The wound is described as having a high microbial load with organisms existing in both planktonic and biofilm states (13, 23, 24). Furthermore, in chronic septic conditions, microbes expand the zone of infection (ZOI) by spreading into necrotic tissue, the smallest host osteons, and within host cells, including fibroblasts, immune cells and the osteocyte lacuno-canalicular network (OLCN). The intra-cellular residence of microbes is an adaptive mechanism making a terrific opponent for the patient and physician (25, 26, 27, 28). These conditions perpetuate microbial survival. In this state, an aggressive debridement is required in conjunction with the use of potent surgical irrigants effective against all aspects of the ZOI: planktonic microbes, EPS and its encased microbes and microbes within small osteons and intracellular-microbial residence, which includes OLCN.

## Wound irrigant mechanics

All irrigants reduce bioburden via the mechanism of dilution, whereby the irrigant fluid continuously and progressively dilutes the microbial load. The goal with the dilutional mechanism is to lower the number of colony forming units below a pathogenic ‘threshold’. Additives in the form of antibiotics, antiseptics (i.e. nonspecific antimicrobial agents), surfactants and buffers provide supplementary antimicrobial action to the lavage, further reducing microbial bioburden. Tactically, the selected irrigant must address the microbial load and biofilm state within the wound. For chemical irrigants, microbial kill requires a threshold irrigant concentration and a minimum time of exposure known as dwell time. It relies on the probability of interaction of the individual microbe and the antimicrobial molecule. If the agent is of high parts per million (PPM) concentration or used for a longer dwell time, the probability of interaction and subsequent microbial kill increases, albeit the cost of host cellular toxicity. Disruption of a biofilm is more complex and requires a multi-modal irrigant to interact with the molecular structures of the EPS, leading to its disruption with exposure of core microbiota, followed by microbial kill. Furthermore, to address the intracellular residence of microbes within host cells, some host cellular toxicity is paradoxically required to liberate and extinguish these intracellular variants. This tactic must be carefully balanced as we emphasize that any irrigant additive exerts collateral host tissue damage, depending on their mechanism, strength and dwell time. All antiseptic agents cause human fibroblast cell death and greatly reduce cellular proliferation required for wound healing. This occurs even at low antiseptic concentrations (29). Moreover, antiseptics also impart the same effect upon immune cells within the wound (30, 31). Toxicity is amplified when multi-modal agents are employed due to synergistic effects. In addition, *in vivo*, these irrigants undergo complex interactions with human tissue and extracellular fluid with their *in vivo* effectiveness muted by host protein binding, ionic sequestration and pH inactivation (1, 32, 33). Therefore, irrigant selection should follow a strategic, graduated escalation in modality number and strength based upon microbial bioload and biofilm state, seeking to balance microbial kill against collateral host damage. A general categorization of irrigants is presented in Table 1. For every irrigant, we discuss their specific properties and mechanism(s) of action and comment upon their tactical use for each orthopaedic wound category.

**Table 1:** Commonly used orthopaedic irrigants in global use.

Number of mechanisms (modes of action)	Primary mechanism of action	Irrigant agents
Single modal	Dilutional	0.9% Normal saline
Dual modal	Dilutional + antimicrobial	0.9% Normal saline + added antimicrobial(s)
Dual modal	Dilutional + non-specific antiseptic	Chlorhexidine Polyhexanide Povidone iodine Hydrogen peroxide Sodium hypochlorite Acetic acid
Multi-modal	Dilutional + non-specific antiseptic(s) + buffer agent + surfactant	1:1 Mixture of 3% hydrogen peroxide and 10% povidone iodine (HP + PI) Bactisure^TM^ Xperience^TM^

## Classification of orthopaedic wound irrigants

### Single modal (dilutional)

#### 0.9% Normal saline (NS)

NS is the most frequently used wound lavage agent in orthopaedic surgery. It is comprised of 0.9% NaCl in sterile, distilled water. Its mechanism involves removal of microbes via a dilutional effect. Microbial load reduction in surgical musculoskeletal wounds ranges 50–70% employing 3–9 litres, and this serves as the baseline in comparing the effectiveness of other wound irrigants (34, 35, 36, 37). NS has no inherent antimicrobial properties. It is used as an isolated agent or as an adjunct to rinsing other irrigants (1). Being isotonic, it is minimally toxic to surrounding host tissues. It is optimal for use in aseptic wounds of low microbial load in planktonic conditions. It can also be used as the preliminary irrigant in septic wounds before the application of a more powerful irrigant. In this technique, the NS irrigant reduces microbial load creating a higher relative ratio for the adjuvant irrigant against residual microbiota. NS alone is appropriate for aseptic wounds with low microbial load and planktonic conditions and for acute septic arthritis when chondrocyte preservation is a priority.

### Dual modal (dilutional + antimicrobial action)

Parenteral antimicrobial agents can be added to saline providing an adjuvant mechanism to the NS irrigant. Historically, bacitracin and polymyxin have been the commonly added antibiotics to saline. Bacitracin is a cell-wall-targeting agent, while polymyxin targets the cell membrane. When used together, they provide a wide spectrum microbial kill. However, these two agents can damage human cell membranes and are locally toxic. Furthermore, these two agents can be systemically absorbed during irrigation, causing host organ toxicity. Given the high risk of nephrotoxicity and anaphylaxis, regulatory bodies globally are discouraging/banning the use of bacitracin in irrigants for safety reasons (1, 38). Other parenteral antimicrobials can be added to NS, but the use of selective antibiotic/antifungals is inefficient, because a single agent (or even a dual combination) will not kill all potential microbes that may exist within the wound (3). Moreover, we caution against the use of targeted antimicrobials as it runs the risk of selecting out resistant microbes, potentially culminating into a more severe infection (21). With no clear published benefits, the World Health Organization (WHO) and the International Consensus Meeting for MSI 2018 (ICM-18) recommend against the use of antimicrobial additives to NS irrigation in prevention and treatment of surgical site infection (1, 39, 40, 41). Hence, the addition of selective antimicrobial additives in NS is discouraged for orthopaedic wound irrigation.

### Dual modal (dilutional + antiseptic action)

#### Chlorhexidine (CH)

CH irrigant is comprised of sterile 0.05% chlorhexidine gluconate (CHG) in 99.95% sterile water commercially available as Irrisept® (Innovation Technologies Inc., USA). CH is a cationic bisbiguanide that ionically binds to microbial cell walls, but its lethal action is primarily at the cell membrane, altering intra-cellular osmotic equilibrium (33). Its uptake by microbes is rapid, within 20 s, with immediate bactericidal action (2). It requires a short dwell time of 2–3 min (42, 43). However, we emphasize that CH’s broad-spectrum biocidal action and ability to disrupt biofilm is seen only with a high concentration of CHG (≥2%), for which there is no commercial agent available (1, 44). At the lower concentrations used in commercial irrigants (≤0.05%), its action is primarily dilutional with a weak bacteriostatic action (1, 33, 44, 45). It has low host toxicity at commercially available concentrations (2). However, CH still remains significantly chondrotoxic even at this low strength (46, 47). Surgeons should be watchful for hypersensitivity reactions and anaphylaxis. It is recommended to rinse the wound with saline after its use even at low concentrations (48). CH is optimal for aseptic wounds with low microbial load and planktonic conditions.

CH has also been combined as 0.015% CH with 1.5 mg/mL Cetrimide in sterile water solution and is available through pharmaceutical companies locally (2). Cetrimide is a quaternary ammonium compound that augments the action of CH by disrupting microbial cell membranes. This combination is essentially tantamount to a low concentration of CH (<0.05%) and is only a weak bacteriostatic agent. This combination is also optimal for aseptic wounds.

#### Polyhexanide

Polyhexanide, also known as polyhexamethylene biguanide (PHMB), is a polymer composed of repeating biguanide units (which is the basic structural unit of CH), linked by hexamethylene chains. The PHMB products are synthetically manufactured and are commercially available as Prontosan® (0.1% polyhexanide with 0.1% betaine, B. Braun, Switzerland) and Preventia® (0.1% polyhexanide with poloxamer, Hartmann, Germany). The polyhexanide molecule binds non-specifically to negatively charged microbial membranes causing increased permeability and leakage of intracellular contents, resulting in microbial cell death. The adjuvant agents betaine (Prontosan) and Poloxamer (Preventia) are added surfactants. It requires a dwell time of 3–5 min (43). *In vitro* studies show effectiveness as a planktonic antimicrobial agent with weak antifungal properties (49, 50, 51). The effective concentration to provide 100% kill of all microbes still requires investigation. Polyhexanide irrigants at 0.1% concentration show effective but incomplete planktonic kill and limited biofilm kill even with their adjuvant surfactant agents. Host cytotoxicity at 0.1% is similar to CH and is also significantly chondrotoxic (43, 52). PHMB irrigants at 0.1% (even with added surfactants) perform similar to 0.05% CH, and PHMB is optimal for aseptic wounds with low microbial load and planktonic conditions.

#### Povidone iodine (PI)

The reported concentrations of PI for orthopaedic wound irrigation are 0.35, 0.5 and 10%. However, the only wound irrigation solution commercially distributed is Surgiphor® (BD, USA), as 0.5% PI in phosphate-buffered sterile saline solution (1, 33, 53). The WHO recommends a dilute, aqueous-PI for wound irrigation at the termination of surgical wounds, espoused by numerous orthopaedic societies (54). PI is a complex of iodine conjugated to polyvinylpyrrolidone, which increases its solubility. PI exerts its antimicrobial action by release of free iodine (*I*_2_), which passes through cell walls and membranes, oxidizing intracellular contents of microbes in a concentration dependent manner with broad-spectrum effectiveness against bacteria, fungi and some spores. It requires a dwell time of 2–5 min for action (1, 43). *In vitro* studies have shown cytotoxic effects of PI even at low concentrations (<0.35%) (55). PI impairs fibroblast viability, affecting wound healing, and is significantly chondrotoxic (56, 57). Furthermore, free iodine can be systemically absorbed, risking toxicity, including acute kidney injury, hypernatraemia, thyroid abnormalities and hypersensitivity reactions (1, 33, 58, 59, 60). We emphasize PI solutions are not effective in completely eradicating planktonic microbes at <10% because free floating planktonic cells must interact with free floating *I*_2_ molecules. In contrast, *I*_2_ readily diffuses through stationary biofilms, oxidizing the EPS and reaching core microbial populations. Its effectiveness in disrupting biofilm increases with higher concentrations (1, 32). In summary, PI at <10% provides incomplete killing of planktonic microbes because of low concentrations (i.e. low PPM of *I*_2_). Dilute PI (0.35 and 0.5%) is optimal for irrigation of aseptic wounds with low microbial load and planktonic conditions.

#### Hydrogen peroxide (HP)

HP is commercially available for medical use at 3% concentration (33). It is a highly reactive oxidizing agent, generating oxygen free radicals within the wound that denatures lipids, proteins and nucleotides, culminating in microbial cell death. The effervescence generated further aids in mechanical debridement. HP also shows synergistic action with CH and PI (61, 62, 63). CH alters the structure of bacterial cell surface to improve penetration of HP. PI with HP create multiple non-overlapping oxidative mechanisms enhancing microbial kill (64). However, the catalase enzyme generated by some bacteria and normal human tissue can mitigate its *in vivo* efficacy (1, 33). HP is cytotoxic to human cells, including chondrocytes (1, 33, 61). The breakdown of hydrogen peroxide into oxygen gas in closed and semi-closed cavities run a risk of air embolism and cardiac arrest, and there are rare cases of embolism reported in orthopaedic surgical wounds (65, 66). HP dwell time is typically 3 min and rinsed with saline afterwards (1, 43, 61). At 3% concentration, it is effective against planktonic microbial states. As a single agent, it is inadequate for biofilm disruption (1, 67). Hence, 3% HP is optimal for acute septic wounds to reduce microbial bioload, accepting potential host toxicity.

#### Sodium hypochlorite – Dakin’s solution (DS)

Sodium hypochlorite irrigant, known as Dakin’s solution (DS), was developed by Henry Dakin during World War I (68). It is readily available commercially or can be mixed by in-house pharmacy. Full-strength DS is 0.5% sodium hypochlorite with a bicarbonate buffer to maintain near-physiologic pH. Full-strength DS is the commonly applied concentration for infected wound treatment (1). It acts by producing hypochlorite ions that non-specifically oxidize microbial cell wall, cell membrane and cytoplasmic structures (33). DS dwell time is 3–5 min (43). It is effective against planktonic microbes with limited efficacy against biofilm states (1, 32, 33, 67). DS is cytotoxic in proportion to concentration and is strongly chondrotoxic (1, 69, 70). Full-strength DS is optimal for use in acute septic wounds with high microbial load and planktonic conditions.

#### Acetic acid (AA)

AA, commonly known as vinegar, is a known antiseptic agent. Currently, it is not sold as a single commercial irrigant. It can be prepared upon request by in-house pharmacy. AA is used at 3% concentration when applied clinically as a single agent for wound lavage. It has several antimicrobial mechanisms related to low pH protonation: microbial membrane disruption, cytoplasmic osmotic stress and intracellular protein unfolding (71). Because of its low working pH, AA is effective against both planktonic and biofilm states; however, a longer dwell time is required, typically up to 20 min (71, 72).

AA has a native pH of 2.5. Clinically, maximal antimicrobial properties of AA are observed at a pH of 4.76 and lower (73, 74). However, *in vivo*, AA undergoes rapid pH deactivation (within minutes), which neutralizes its antimicrobial activity (75, 76). For sustained effectiveness, AA must have an adjuvant buffer (i.e. sodium acetate) maintaining a low wound pH for adequate antimicrobial kill during its dwell time (43). AA also imparts its toxic effects to host tissues, killing fibroblasts and immune cells. Its acidity makes it chondrotoxic (77, 78). AA as a single agent (i.e. without a buffer) is optimal for acute septic wounds with high microbial bioload. For biofilm disruption, AA is better suited when combined into a multi-modal commercial irrigant where a buffering agent optimizes therapeutic use (see the Multi-modal section).

### Multi-modal (dilutional + antiseptic(s) + surfactant + buffer)

#### 1:1 Hydrogen peroxide and povidone iodine (HP + PI)

A 1:1 combination of 10% PI with 3% HP (HP + PI) is prepared at the time of surgery in the operating room using new, single-use, sealed, commercially available bottles of each product (4 oz/118 cc each). The bottles are mixed at the time of use with repeat mixes used as necessary with new bottles. HP + PI is readily used globally and has been used for orthopaedic wound lavage for over two decades (79). The dwell time is typically 3–5 min (43). HP + PI has multiple synergistic mechanisms, creating non-overlapping oxidative mechanisms that effectively disrupt biofilm EPS and enhance microbial kill (64). HP reacts with iodine via a redox reaction, forming iodide (I−). Iodide then accelerates the decomposition of hydrogen peroxide, triggering the formation of hypoiodite, superoxide and hydroxyl free radicals. This synergistic production of reactive oxygen species damages microbial cell membranes, proteins and DNA, resulting in microbial death (32, 80). Host toxicity is similarly synergistic with significant chondrotoxicity. HP + PI is highly effective against both planktonic and biofilm states, including fungal species (32, 80, 81, 82). To date, it has been the most cost-effective agent among multi-modal anti-biofilm irrigants. HP + PI is optimal for chronic septic wounds with biofilm, including fungal microbes.

#### Bactisure^TM^ (BS)

Bactisure™ wound lavage solution (Zimmer-Biomet, USA) is a commercially available acetate-based irrigant. The ingredients and mechanisms in BS are listed in Table 2 (3). Being a powerful non-specific biocidal, it requires a post-irrigant rinse with saline after 5 min (43). The recommended dwell time is 3–5 min. *In vitro* studies have demonstrated high fibroblast toxicity even at a brief exposure time of 1 min, which can adversely impact wound healing (78). It is strongly chondrotoxic (83). BS has broad-spectrum efficacy against bacterial and fungal species in both planktonic and biofilm states (3, 84). Because of its high cytotoxicity profile, BS is best suited for chronic septic wounds with an established biofilm. Future studies are required to show clinical effectiveness in longitudinal comparison with other biofilm-disrupting agents.

**Table 2:** Ingredients of Bactisure^TM^ wound irrigant.

Ingredient	Weight-to-volume ratio	Mechanism of action
Acetic acid	5%	- Microbiota biocide in planktonic and biofilm states
		- Biofilm dissolution
		- Microbial membrane disruption
		- Intracellular protein unfolding/denaturation
		- Cytoplasmic osmotic stress
Sodium acetate	3%	- Buffer agent: keeps acetic acid in the active antibiocidal range
		- Hygroscopic salt: imparts osmotic stress to microbiota
Ethanol	10%	- Surfactant: emulsification of biofilm matrix
		- Rapid disruption of cell membrane function by protein denaturation and dissolution of lipid membranes
Benzalkonium chloride	0.13%	- Cationic surfactant: emulsification of biofilm matrix
		- Disruption of microbial membrane integrity
		- Denaturation of cytoplasmic proteins
Water	Q.S.* 1L	- Osmotic stress
		- Mechanical + dilutional agent when used with pulsed mechanical lavage

* Quantum satis – ‘the amount which is enough’.

#### Xperience^TM^ (XP)

Xperience™ wound lavage solution (Next Science™, USA) is a commercially available citrate-based irrigant. The ingredients and mechanisms in XP are listed in Table 3 (85). It has the advantage of not requiring a saline rinse after application (86). *In vitro* studies have demonstrated its efficacy in both planktonic and biofilm conditions (86). Although there is not a stated dwell time, exposure of XP for at least 3–5 min is needed to exert its antimicrobial and biofilm-disrupting effects. Compared to HP + PI and BS, XP has lower antimicrobial effectiveness with a lower host toxicity profile due to its lower ingredient concentrations by design (32, 85). XP is acceptable for treating chronic septic wounds that do not involve fungal microbes. Future clinical studies are required to compare XP effectiveness to other biofilm-disrupting agents (87).

**Table 3:** Ingredients of Xperience^TM^ wound irrigant.

Ingredient	Weight-to-volume ratio	Mechanism of action
Citric acid	3.25%	- Microbiota biocide in planktonic and biofilm states
		- Biofilm dissolution
		- Microbial membrane disruption
		- Cytoplasmic protein unfolding/denaturation
Sodium citrate	3.13%	- Buffer agent: keeps citric acid in the active antibiocidal range
Sodium lauryl sulphate	0.10%	- Surfactant: reduces surface tension of biofilm and lyses microbial cells
Water	Q.S.* 1L	- Osmotic stress
		- Mechanical + dilutional agent when used with pulsed mechanical lavage

* Quantum satis – ‘the amount which is enough’.

## Irrigation method and volume

The method of irrigant delivery and selected volume are integral factors to wound lavage. The three established lavage methods for open orthopaedic procedures include i) continuous gravity flow irrigation (GFI) using large bore tubing or pouring from an irrigation container directly onto the wound, ii) syringe/bulb irrigation (SBI) using 20–100 cc syringes/bulbs and iii) mechanical pulsed irrigation (MPI) via a battery powered device. Table 4 summarizes the characteristics of each delivery method. The difference between the three methods is the impact pressure to host tissues and the depth of fluid penetration. Method selection depends upon three main factors: the extent of foreign debris contamination, microbial bioload and microbial state (i.e. presence of an established biofilm). Microbes adherent to tissue surfaces (not yet in a biofilm state) are dislodged with irrigant pressures ≥8 psi (88). Embedded foreign debris requires pressures >15 psi to dislodge materials (89). Tissue damage with cellular necrosis begins with impact pressures ≥20 psi (90).

**Table 4:** Characteristics of irrigant delivery.

Irrigation method	Generated pressures	Mechanism of bioload reduction	Advantage	Disadvantage	Host tissue damage	Best use
Gravity flow (GFI)	0.5–2 psi*	Dilutional	Easy	Does not remove adherent, sessile microbes on tissues	None	Planktonic microbial conditions with no foreign debris load
			Inexpensive			
Syringe/bulb (SBI)	2–11 psi	Dilutional + some mechanical	Inexpensive	Time-consuming when delivering large volumes	Mild	Planktonic microbial conditions with low foreign debris load
			Dislodges adherent surface microbes (not in biofilm)			
Mechanical pulse (MPI)	10–80 psi	Dilutional + mechanical	Deep penetration	Incurs host tissue damage	Significant	High foreign debris load
				Unintended propulsion of microbes into deeper wound areas		Chronic infection with entrenched biofilm

* psi, pounds per square inch.

With GFI, the irrigant is delivered from an irrigant bag to the wound via tubing with an inner diameter typically 8–10 mm. Tissue impact pressures are low, approximating 0.5–2 psi (88, 91, 92). This method is considered atraumatic to host tissues and is inexpensive. The mechanism of bioload reduction is dilutional with pressures inadequate to dislodge microbes from tissue surfaces (88). With SBI, the typical exit diameter of the bulb/syringe is 4–5 mm, generating peak pressures ranging from 2 to 11 psi (93). This method is inexpensive, but time-consuming if applying large irrigation volumes. The mechanism of bioload reduction is dilutional with some mechanical disruption. Bulb/syringe pressurization generates peak pressures that can dislodge microbes from tissue surfaces. With MPI, the generated pressures are significantly higher, ranging from 10 to 80 PSI with nozzle exit diameters of 1.5–2.5 mm (94, 95). The mechanism of bioload reduction is dilutional and hydromechanical with pulse pressures that can separate adherent microbes from host tissues, dislodge entrenched foreign debris and mechanically disrupt the EPS matrix of biofilm (36). MPI is time efficient, allowing directed distribution of large irrigant volumes in a relatively short period of time (36). With MPI, the irrigant fluid can penetrate as deep as 15.6 mm and can also reach within osteons when directly applied over bone (95, 96, 97, 98). This method damages host tissues (90, 98). Furthermore, through hydro-dissection, microbiota can be paradoxically pushed into deeper spaces within the wound (94, 97, 99). Finally, the cost of battery-powered mechanical devices is significant, and in financially constrained healthcare systems, this method is infrequently used. High-pressure MPI should only be used with NS, as other irrigants have prohibitive toxicity profiles. Low-pressure MPI (<15 psi) has been cautiously utilized with volumes no greater than 1 L. These include dilute PI, XP and BS (100).

The selected irrigation volume relates to bioload conditions. When using the irrigant primarily for dilutional effect, the volume that maximizes microbial load reduction depends on wound size, microbial load and state, emphasizing there will be no reduction of microbes within a biofilm (84, 96). The literature lacks evidentiary support for an optimal minimum irrigant volume (101, 102). Heuristic recommendations are for using an irrigant volume of 6–9 L (101, 103). Smaller wound areas practically require less volume. For chronic infections with biofilm, a multi-modal irrigant is needed to disrupt the EPS; however, toxicity and cost become limiting factors. Tactically, a practical approach is MPI with NS of 6–9 L prior to the application of a multi-modal agent, followed by post-lavage NS rinse of 3 L.

Taking into consideration risk, benefit and cost parameters, GFI and SBI are suitable for aseptic wound lavage with irrigation volumes of 1–3 L. For acute septic wounds, SBI with irrigant volumes of 6–9 L is better suited to reduce planktonic microbial load and address adherent tissue microbes. Finally, MPI with volumes of 6–9 L is optimal to address wounds with high foreign body load and chronic septic conditions with a biofilm.

## Conclusion

We advocate against, as common practice, the ‘one size fits all’ use of a single irrigant product for all open orthopaedic procedures. This approach, which provides the surgeon simplicity and comfort, can be at times inadequate to address the task at need, or at times can result in unnecessary local tissue toxicity. Instead, irrigant selection should follow a tactical multi-modal escalation based upon microbial bioload and biofilm state. Single-modal dilutional lavage (e.g. saline) or select dual-modal agents are adequate for aseptic orthopaedic wounds of low microbial planktonic load. For acute septic conditions, at minimum, an effective dual-modal agent is advocated to address the high microbial planktonic bioload. For chronic septic conditions, multi-modal agents are required to disrupt the biofilm EPS matrix and kill microbes. The recommended usage of each irrigant product is summarized in Table 5. The method of irrigant delivery and volume used is an integral factor in wound lavage and should also be selected based on bioload conditions and state (i.e. biofilm). The principles of irrigant selection presented herein can be applied to novel future irrigants if the mechanism(s) of action, efficacy and effective concentrations of the irrigant are clearly identified. In the future, we anticipate additional lavage agents to be introduced, but it will be limited because any new irrigant must be able to eradicate the broad spectrum of encountered microbes and be tolerable to the human host. Future studies will be required to assess the cost–benefit of new products to justify their use against equally efficacious cheaper alternatives in present use.

**Table 5:** Recommended wound irrigant applications.

Aseptic	Acute septic	Chronic septic (biofilm disruptors)
0.9% normal saline	0.9% normal saline*	1:1 mixture (HP + PI)
Chlorhexidine (0.05%)	Hydrogen peroxide (3%)	Bactisure™
– Irrisept®		
Polyhexanide (0.1%)	Sodium hypochlorite (0.5%)	Xperience™
– Prontasan®	– Dakin’s full strength	
– Preventia®		
Povidone iodine (0.5%)	Acetic acid (3%)	
– Surgiphor®		

* 0.9% normal saline should be used for acute septic arthritis when chondrocyte preservation is a priority.

## ICMJE Statement of Interest

The authors declare that there is no conflict of interest that could be perceived as prejudicing the impartiality of the work reported.

## Funding Statement

This work did not receive any specific grant from any funding agency in the public, commercial or not-for-profit sector.
